# A Review of Machine Learning Applications in Mechanical Metamaterial Design

**DOI:** 10.3390/ma19132766

**Published:** 2026-06-30

**Authors:** Galymzhan Turysbekov, Ulanbek Auyeskhan, Andrei Yankin, Asma Perveen, Didier Talamona

**Affiliations:** 1 Department of Mechanical and Aerospace Engineering, School of Engineering and Digital Sciences, Nazarbayev University, Astana 010000, Kazakhstan; galymzhan.turysbekov@nu.edu.kz (G.T.);; 2 Samsung Electronics Co., Ltd., Seoul 005930, Republic of Korea; ulan.auyeskhan@gmail.com

**Keywords:** mechanical metamaterials, machine learning, inverse design, data-driven design, finite element method, generative models

## Abstract

**Highlights:**

- Integrating Machine Learning with metamaterial design enables the development of innovative structures with unprecedented properties, applying different ML architectures.
- Machine Learning techniques can significantly accelerate the design cycle, generating novel designs using inverse design frameworks.
- Emerging trends toward multifunctional materials and autonomous discovery systems show strong potential for addressing challenges related to interpretability and manufacturability.

**Abstract:**

Mechanical metamaterials are architected materials that exhibit unusual mechanical properties arising from their internal geometry. This paper reviews recent developments in the application of machine learning for the design and analysis of these structures. It categorizes common architectures, including strut-based lattices and triply periodic minimal surfaces, and details the end-to-end design workflow, from dataset preparation and preprocessing to the iterative, simulation-based validation approach. The review compares a range of model architectures. These include foundational models like deep neural networks, fully connected and convolutional neural networks, graph neural networks, and generative models such as GANs and diffusion models. Applications in mechanical property prediction and inverse design are highlighted with examples using finite element simulations and generative design models. A structured design workflow and a comparative summary of recent studies are presented to guide future research and application. This review aims to support the development of ML frameworks for next-generation metamaterial design.

## 1. Introduction

Mechanical metamaterials represent a groundbreaking class of engineered materials distinguished by their unique internal structures, which enable them to exhibit extraordinary mechanical properties not typically found in natural materials [1,2,3]. The concept is rooted in the manipulation of their architecture rather than their chemical composition, leading to innovative designs that can achieve desired mechanical responses through careful engineering [4,5,6,7]. These materials are meticulously designed with precise geometrical arrangements at the micro- or nanoscale, allowing them to demonstrate unusual behaviors such as negative Poisson’s ratios (expanding laterally when stretched) [8], tunable stiffness, negative compressibility, distinct orthotropic properties, and negative thermal expansion [9,10,11,12,13,14,15]. Additionally, they can achieve a high strength-to-density ratio, providing exceptional strength while remaining lightweight—qualities that make them particularly appealing for applications in aerospace, automotive, and biomedical fields [16]. As manufacturing technologies continue to advance, especially with the growing use of additive manufacturing (AM), researchers are now exploring increasingly complex architectures, realizing sophisticated concepts that were previously considered unattainable [4].

Numerous architectural types of metamaterials have been developed and explored along with their properties and variations. Those structures can be classified into groups of strut-based, curve-based, chiral, pentamode, rotating polygon, honeycomb, kirigami, and others [17,18,19,20].

Strut-based metamaterials are a foundational class, consisting of cylindrical struts interconnected at nodes to form lattice frameworks [21,22,23]. Strut-based structures include Body-Centered Cubic (BCC), Face-Centered Cubic (FCC), octet truss, tesseract lattices, and others [24,25,26]. Even more variations can be derived by hollowing the struts [27] or by combining different lattice types into hybrid structures [28,29,30].

Curve-based metamaterials represent another large structural group that can be fully defined by mathematical functions, encompassing both 2D parametric curves and 3D surfaces [31]. A prominent category is Triply Periodic Minimal Surfaces (TPMSs), which are mathematically defined structures that repeat periodically in three-dimensional space and possess zero mean curvature at every point. Common examples of TPMS include the gyroid, Schwarz Diamond, Primitive, and Neovius structures [31]. Some studies combine struts and TPMS structures to design another variation of metamaterials [21].

Other notable architectures include pentamode metamaterials, which consist of slender rods arranged in a specific geometric configuration to create highly anisotropic mechanical responses [32]. Adjusting the length-to-diameter ratio of the rods can influence the properties of the structure, where longer and thinner rods result in lower shear stiffness while maintaining bulk stiffness. Further designs, such as chiral, rotating polygons, and honeycomb structures, also contribute to the vast design space, each offering unique mechanical behaviors.

**Figure 1:**
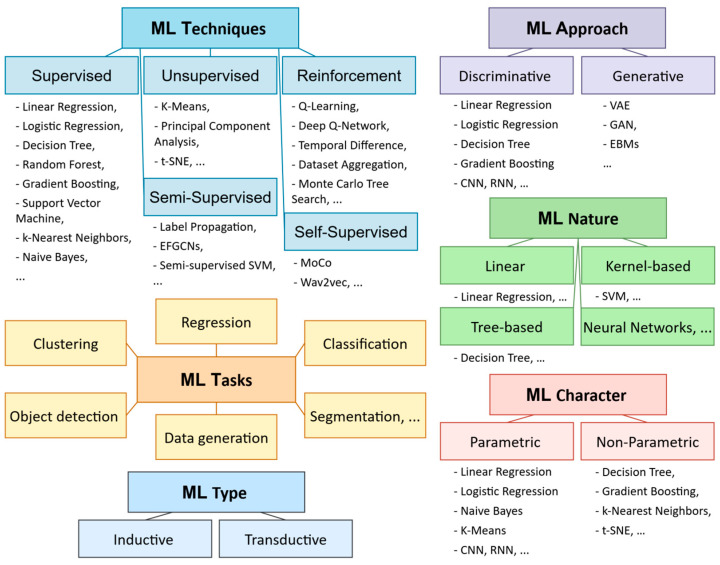
ML categorization.

Machine Learning (ML) is a subset of artificial intelligence that focuses on developing algorithms that learn from data and improve their performance over time without explicit programming. By identifying patterns within datasets, ML models can make predictions, recognize trends, and solve complex problems [33,34]. ML algorithms can be categorized based on their learning techniques, their structure, task type, and so on [35,36,37]. Figure 1 summarizes these categories.

ML techniques include supervised, unsupervised, reinforcement, and semi-supervised learning. Supervised ML uses labeled inputs and outputs to train a model that predicts the output for new inputs, whereas unsupervised ML uses unlabeled data for learning patterns. Reinforcement ML trains an agent to maximize rewards via interaction with the environment and feedback from actions [38]. Semi-supervised ML combines a small number of labeled data with a large number of unlabeled data, providing higher accuracy compared to unsupervised ML and requiring less labeling compared to supervised ML [39]. Self-supervised ML uses unlabeled data for the generation of “pseudo-labels” for training without human annotation, and it is sometimes regarded as a branch of unsupervised ML [40,41]. Meanwhile, ML can be categorized by different tasks: regression, classification, clustering, object detection, segmentation, data generation, among others [36]. These paradigms provide the basis for the selection of appropriate model architectures, as discussed in Section 3.2.

The ML approach can be discriminative or generative. The discriminative approach (common for most ML models) focuses on the target without modeling how the data was generated, whereas the generative approach uses assumptions about how the data was generated [42]. Another difference is that discriminative models use labeled data (common in supervised learning), whereas generative models usually utilize unlabeled data [43].

Model character can be parametric or nonparametric. In parametric models, the number of parameters is fixed (finite) and does not change with new data. In nonparametric models, the number of parameters is not fixed and can grow with additional data [37].

Learning type can be inductive or transductive. Transductive learning predicts specific domain examples for reasoning without generalization, unlike inductive learning. An example of a transductive model is the KNN, which utilizes the training data at each prediction instead of modeling from it [44].

Finally, model nature/type: linear models (e.g., Linear Regression), kernel-based models (e.g., Support Vector Machine), tree-based models (e.g., Decision Tree), neural networks, etc. [45].

In recent years, various ML techniques and methods have become an essential tool in the design and analysis of metamaterials. The design of such materials often involves navigating high-dimensional parameter spaces where traditional methods can be inefficient and computationally expensive [46]. ML models offer a transformative approach by learning the underlying relationships between structure and function from data, enabling rapid predictions, efficient optimization, and inverse design. This approach significantly accelerates the design cycle and reduces reliance on extensive simulations. For instance, generative ML models have been employed to rapidly design metamaterials with prescribed mechanical behaviors, achieving high fidelity between target and experimentally measured results [47]. Furthermore, ML techniques have been utilized to design and optimize thermal metamaterials, enhancing their performance and functionality [48], and have been applied to phononic metamaterials to develop materials with tailored acoustic and elastic properties [49]. These examples underscore the growing importance of ML in metamaterials design, enabling the exploration of larger design spaces, reducing computational costs, and fostering the discovery of novel material configurations with desired properties.

As ML is increasingly being integrated into research, studies have attempted to systematize its application. Current reviews cover AI applications across a wide range of metamaterials. They can include acoustic, photonic, electromagnetic, and other systems. Additionally, specialized reviews often focus on specific materials, including their manufacturing constraints. Comprehensive review papers are presented in [1,17,20,46].

By contrast, rather than a broad review, this paper addresses the challenge of translating metamaterial geometries into formats that ML can process. This work analyzes how these different geometric representations influence the choice of ML models for tasks like forward prediction of complex structural performance and inverse design. This review provides the integration of structural, data, and algorithmic components into a practical, decision-making framework for applying ML to solve complex metamaterial design problems.

Various structural types are covered, such as strut-based lattices, triply periodic minimal surfaces, and other structures. It also incorporates methods for dataset preparation, simulation-based data generation, and preprocessing techniques. Supervised, unsupervised, reinforcement, and semi-supervised learning methods are examined, highlighting their roles in property prediction, optimization, and inverse design. In addition, a comparative analysis of recent studies is provided, along with a taxonomy that links ML models to specific mechanical tasks. A guide for future research and practical applications in the field of metamaterial design is provided.

## 2. The Machine Learning Workflow for Metamaterial Design

The application of ML to metamaterial design is not a single step but a comprehensive, iterative process. This section outlines the typical workflow, from initial data handling to the final design verification, providing a practical framework that connects the various concepts discussed in this review.

### 2.1. Dataset Preparation and Representation

**Figure 2:**
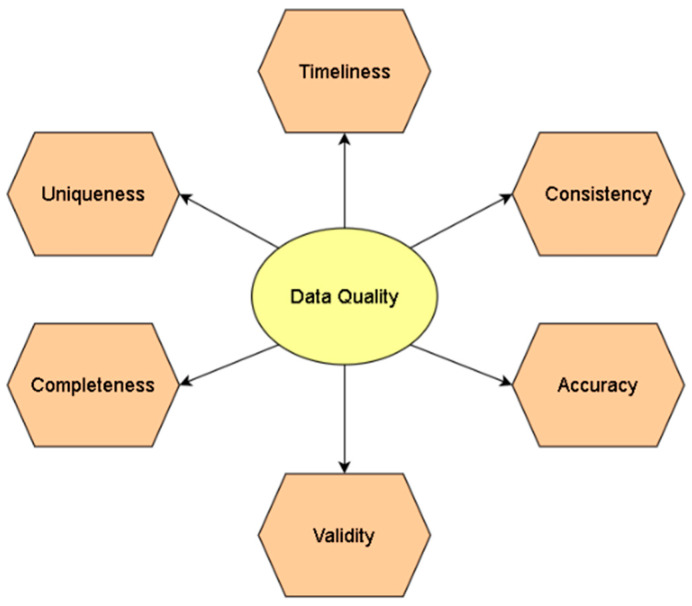
Data quality.

The foundation of any ML-driven approach lies in the quality of the used datasets (Figure 2), such as consistency, accuracy, completeness, uniqueness, timeliness, and others [50,51,52,53]. Specifically, consistency means the utilization of uniform representations and formats throughout a dataset; completeness refers to whether there is enough data to deliver robust ML results; accuracy signifies whether the data are correct and error-free; timeliness refers to whether the data are updated and relevant; and uniqueness means the absence of duplicates.

Datasets serve as the basis for ML models to identify patterns, create new structures, predict mechanical properties, and solve inverse design problems. A well-prepared dataset provides reliable performance in a variety of applications, including generative design and predictive analysis [54]. This section focuses on preparing datasets that encapsulate these features. Such datasets must comprehensively represent the diversity of design, material characteristics, and performance metrics to maximize the effectiveness of different models.

Datasets can originate from several sources. Firstly, data can be obtained from existing datasets or literature. There are extensive materials databases, such as AFLOW [55,56], Materials Project (MP) [56,57], MATDAT [57], MatWeb [58,59], MatMatch [59], and MatNavi [60], which contain vast amounts of material properties obtained from experimental measurements and first-principles calculations, including mechanical properties like elastic constants, tensile strengths, fracture toughness, and hardness [61]. Additionally, the other option is to filter and collect sub-datasets of specific properties or material classes through these platforms. Text processing techniques, like Natural Language Processing (NLP), enable automated workflows for extracting features from journal articles [61]. For example, an NLP-based approach trained on over 640,000 materials synthesis articles has facilitated the creation of synthesis parameter databases, broadening ML applications in material discovery. Recent advancements include Mechanical MNIST, a benchmark dataset derived from bitmap images, labeled with mechanical responses calculated from finite element methods (FEA) simulations, to evaluate heterogeneous material models [61].

**Figure 3:**
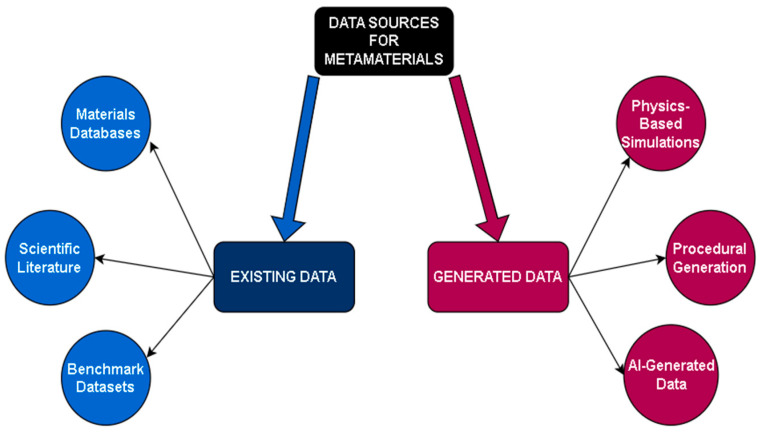
Possible data sources for metamaterials.

Datasets can also be generated based on specific design spaces, which are considered as Simulated Data (or Generated Data). These datasets can include features and distributions controlled by domain knowledge in materials science and mechanics [61]. Different software tools may help generate databases based on users’ needs. For example, FEA has been used to create datasets of 3D microstructures in high-contrast composites, 2D tessellated composites, and metamaterials. Synthetic microstructure images can also be generated using techniques like Gaussian random fields (GRFs) [61], as well as advanced deep learning models capable of text-to-image synthesis [62], systems-level design [63], and predictive image generation [64]. Possible data sources are illustrated in Figure 3.

Geometries for mechanical metamaterials can be modeled using “bottom-up” or “top-down” approaches. The bottom-up method builds structures from basic geometric elements, while the top-down approach removes material to create cellular structures [65]. Modeling parameters, pixels/voxels, and meshes represent these geometries, balancing computational efficiency and design diversity. For instance, voxel-based representations are suited for convolutional neural network (CNN) models due to their compatibility with convolutional layers, which makes them suitable for volumetric optimization [65,66]. However, they can cause staircasing artifacts in boundaries that require significant refinement to achieve smooth geometries [66]. Meanwhile, meshes for FEA simulations provide high-fidelity representations of 3D structures, but require significant memory and computational resources [65,66].

Graph representations extend convolutional approaches. For instance, such a method can be applied efficiently for capturing structure–property relationships of truss lattices by representing the connections of nodes and struts [67,68]. However, it might encounter more challenges in representing more complex geometries such as TPMS [69]. Hereby, the selection of an appropriate geometric representation is a crucial step. It can affect not only the design types explored by ML but also the accuracy and physical validity of the predicted mechanical behavior.

### 2.2. Dataset Preprocessing

Preprocessing is a vital step to prepare raw data for ML applications. ML models often expect data in structured formats such as images, graphs, or text [61]. Data preprocessing includes data cleaning, data integration, data transformation, and data size adjustment [70,71,72].

Data cleaning involves handling missing values and noisy data, as well as removing duplicates, irrelevant data points, and outliers that can decrease model performance [73,74,75]. Missing values might be addressed using various techniques, from simple approaches such as dropping data or utilizing mean/median imputations to more advanced methods based on ML algorithms [76].

Data integration combines information from different sources into a single dataset. Proper integration may help to decrease redundancies and inconsistencies, enhancing the overall process accuracy and speed [72].

Data transformation encompasses data generalization, feature scaling, distribution transformation, and handling categorical values. Data generalization replaces low-level values with higher-level ones [77]. Data scaling methods (e.g., min–max normalization, z-score standardization) rescale the original values into comparable ranges, whereas distribution transformations (e.g., logarithmic transformation) decrease skewness and make the data more symmetric [70]. Also, categorical variables often should be converted into numerical ones, for example, when encoding post-processing labels such as heat treatment after manufacturing.

Data size adjustment is utilized when the dataset is either too large or too small. Data reduction decreases the data volume, minimizing loss in predictions. Reduction can be used to input features, dimensionality reduction (e.g., principal component analysis (PCA)), or to the number of samples, numerosity reduction (e.g., data aggregation). Conversely, feature engineering adds new features (e.g., squared or cubic terms) into the dataset to improve model performance. Another approach is data augmentation, which includes techniques as rescaling, cropping, flipping, and/or adding random noise [70,71,72]. These methods are especially useful when dealing with limited samples, as they increase the dataset size. Thus, data augmentation helps to improve the robustness of ML models [61].

### 2.3. The Design and Verification Loop

**Figure 4:**
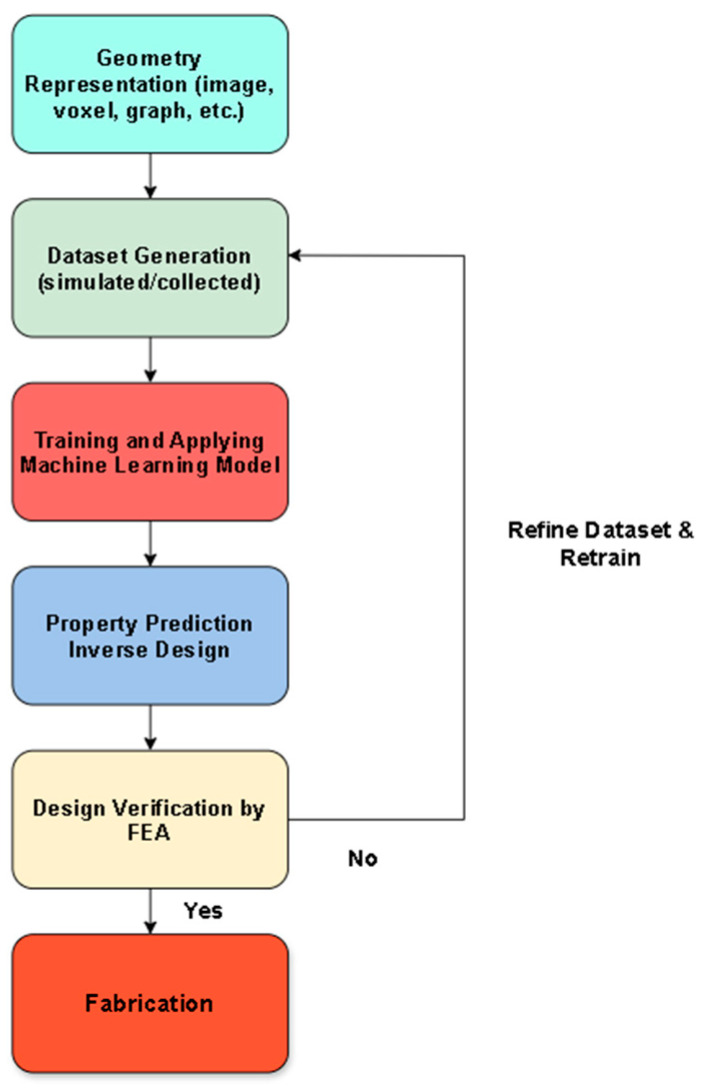
Overview of the ML workflow for mechanical metamaterial design.

The ML workflow is best understood as a closed-loop, iterative process that integrates data-driven design with physics-based validation. This synergy is crucial for creating physically plausible and high-performance metamaterials. The process, often illustrated as a cycle, begins with the dataset steps described previously and proceeds as illustrated in Figure 4.

First, ML models are trained and applied to either predict material properties (forward design) or generate novel structures based on target performance criteria (inverse design). Recent works have employed neural operator transformers and diffusion models to enable such inverse design of metamaterials with complex nonlinear mechanical responses, using signed distance functions or field-based representations [78,79]. Physics-guided ML strategies and generator-forward surrogate architectures have also been used to ensure that generated structures obey underlying mechanics laws [80,81].

However, the designs proposed by these models are not immediately accepted. They must first be validated to ensure their real-world performance will match the prediction. The most common and critical step in this validation phase is the use of high-fidelity numerical simulations, most notably the FEA. For instance, several generative models propose novel 3D metamaterial architectures, which are then immediately simulated with FEA to verify their mechanical behavior, such as their stress–strain response or auxetic properties [79,82].

This verification step is not just a final check, but also the core of the iterative workflow. If the FEA results show that a design fails to meet the desired criteria, the new, high-fidelity data point (i.e., the ML-proposed geometry and its corresponding FEA-calculated properties) is fed back into the training dataset. This refinement process, where the model learns from its own “mistakes”, systematically improves the model’s accuracy. Some advanced workflows employ hybrid or evolutionary algorithms accelerated by neural networks, allowing quick updates to design populations and better alignment with nonlinear design goals [82]. Some workflows also incorporate active search and generative pipelines, where ML-evolutionary algorithm frameworks propose and evolve metamaterial designs, with each candidate tested in simulation and optimized accordingly [83].

This iterative cycle of ML prediction, FEA validation, and data feedback continues until a design is generated that both meets the target specifications and is confirmed to be physically robust. Once a structure is fully verified through this loop, it can proceed to the final stage: physical fabrication, typically using AM techniques to realize the complex geometries [82,83]. This framework seamlessly integrates the design exploration capabilities of ML with the physical accuracy of simulation-based validation, establishing it as a foundational approach in metamaterial design.

## 3. Overview of ML Techniques in Metamaterial Design

The successful implementation of the design workflow described in Section 2 depends on the selection of an appropriate ML model. Different architectures offer distinct strengths and are better suited to specific data representations and mechanical metamaterial design tasks [61]. This section provides a detailed overview of common ML models employed in mechanical metamaterial design.

### 3.1. Framework for Comparison: Overview of Tables

To facilitate a clear and structured comparison of existing ML applications in metamaterial design, this review summarizes findings from a wide range of recent studies. A detailed, quantitative summary of these studies is provided in Appendix A Table A1, which outlines the specific models, datasets, tasks, and performance outcomes for each work. Additionally, a qualitative analysis of the advantages and disadvantages of each major model architecture is presented in Appendix A Table A2.

Table A1 provides an overview of different architectures employed in the literature. They range from foundational models like Deep Neural Networks (DNNs) and their common variants like Fully Connected Neural Networks (FNNs) and Multi-Layer Perceptrons (MLPs), to more specialized architectures such as Convolutional Neural Networks (CNNs) and generative models like Generative Adversarial Networks (GANs). The corresponding “ML Task” column reveals that these models are mostly applied to three core objectives: forward prediction (linking structure to properties), inverse design (generating structures from target properties), and optimization. In this context, optimization refers to the process of finding the optimal design parameters that maximize or minimize a certain performance metric. For example, finding the structure with the highest possible stiffness-to-weight ratio. A notable trend is the combination of these tasks into integrated, multi-stage workflows.

Additionally, Table A1 presents the datasets used across the reviewed studies, revealing the strong dependence on computationally generated data. Most works use physics-based simulation tools, typically the FEA for mechanical analyses, and methods such as Rigorous Coupled Wave Analysis (RCWA) or Finite-Difference Time-Domain (FDTD) simulations for other responses. The scale of these datasets, as listed in the table, varies significantly, ranging from just a few hundred to over one hundred thousand samples. This highlights the varying data requirements across models and tasks.

Also, quantitative performance in these studies is typically assessed using various metrics such as Mean Squared Error (MSE), Mean Absolute Error (MAE), and the Coefficient of Determination (R^2^), which provide a consistent basis for evaluating model accuracy. Overall, this table offers a comparative look at how different ML models are being applied to similar or related problems.

Table A2 offers a more detailed summary of the different ML model architectures. It generalizes the findings from the literature to outline the primary advantages and disadvantages of each class of model. This contributes to a clearer understanding of the strengths and constraints of each model and their alignment with specific metamaterial design goals, while also streamlining the comparison process to identify the most effective architecture for a particular application.

Together, these tables provide a robust framework for understanding and evaluating the role of different ML models in advancing the field of mechanical metamaterials.

### 3.2. Common Machine Learning Models in Metamaterial Design

The models used in metamaterial design can be categorized in two primary ways: by their learning paradigm and by their specific architecture. The choice of paradigm dictates how the model learns from data. Supervised learning is the most common approach, where a model is trained on a labeled dataset to map inputs (e.g., structural parameters) to known outputs (e.g., mechanical properties) [84]. This is highly effective for tasks like property prediction and classification due to its accuracy and implementation [61,65]. In contrast, unsupervised learning works with unlabeled data to identify inherent patterns or groupings, which is useful for dimensionality reduction or discovering novel design families [84]. Other paradigms gaining traction include Reinforcement Learning, where an agent learns to make optimal design decisions by interacting with an environment to maximize a cumulative reward [85], and Semi-Supervised Learning, which uses a mix of labeled and unlabeled data, a practical approach when data labeling is expensive [61]. Table 1 summarizes the characteristics, predictive performance, and implementation feasibility of each learning paradigm relevant to metamaterial design. Black and white boxes represent the rating of each paradigm in terms of property prediction performance and implementation feasibility. A higher number of filled squares indicate better performance or feasibility, and white squares represent lower ratings.

Building on the paradigms outlined in Section 1, this section focuses on the model architectures commonly applied in mechanical metamaterial design. These paradigms are implemented through various model architectures, with a strong emphasis on Deep Learning (DL). DL is a subset of ML characterized by multi-layered neural networks capable of learning complex, hierarchical patterns directly from data [33]. As demonstrated in Table A1, the recent literature shows that the field mostly depends on Deep Neural Networks (DNNs), often implemented as a Fully Connected Neural Network (FNN) or Multi-Layer Perceptron (MLP). These networks, composed of interconnected layers of neurons that apply weighted transformations and nonlinear activation functions, are versatile tools for both forward prediction and inverse design tasks [65]. For example, they have been used to tune the bandgaps of lattice metamaterials [86] and to predict the optical properties for the free-form inverse design of metasurfaces [87]. Moreover, DNNs are widely applied because of their flexibility and power in capturing the complex and nonlinear relationships between metamaterials’ geometric parameters and their resulting mechanical properties [88,89]. While their flexibility is a major advantage, DNNs often require large, high-quality datasets to train effectively and can be prone to overfitting [90]. Furthermore, modern techniques can allow DNNs to achieve high accuracy even with limited training data, for instance, by using data-enhanced iterative few-sample learning algorithms, which mitigates the need for extensive initial simulations [91].

Building upon the basic FNN structure, more specialized architectures have been developed to handle the specific data types common in metamaterials. CNNs are designed for grid-like data, using convolutional filters to automatically extract spatial features, which removes the need for manual feature engineering [92,93,94]. This makes them suitable for processing 2D images or 3D voxel-based representations of microstructures, which are common outputs of FEM simulations [65]. Graph Neural Networks (GNNs), on the other hand, are tailored for non-Euclidean data and are suited for representing strut-based lattices as a collection of nodes (joints) and edges (struts), allowing them to learn directly from the material’s topology [61].

In contrast to predictive models, generative models are central to the progress of automated inverse design, with a primary goal of creating new material structures that exhibit target properties. Variational Autoencoders (VAEs) are widely used to generate new, complex metamaterial designs, and have been used in workflows for capturing the complex relationships of smart radiation devices [95]. GANs use a competitive two-network structure (a generator and a discriminator) to produce high-quality, realistic designs [65,96]. These generative approaches, as demonstrated in several studies, are highly effective for exploring vast design spaces and discovering novel architectures that meet specific performance criteria [82,83].

ML applications in metamaterial design have advanced from simple single-objective tasks to integrated, multi-stage processes. Although the core tasks continue to be forward prediction and inverse design, Table A1 highlights a notable trend toward integrating these tasks within hybrid frameworks. A common way is to train a fast and accurate forward model, like an FNN or CNN, to act as a surrogate for computationally expensive simulations [97]. This model is then integrated into an optimization framework, typically driven by evolutionary algorithms like Genetic Algorithms (GAs), to efficiently explore the design space and solve the inverse problem [98]. This approach creates a workflow that uses the predictive power of ML to accelerate optimization and the discovery of advanced metamaterials.

For forward prediction tasks, where mechanical properties are predicted from known structural features, supervised models like CNNs and DNNs are highly effective. For the goal of inverse design, generative models such as VAEs and GANs are indispensable for creating novel designs and exploring innovative configurations. Meanwhile, architectures like GNNs and paradigms such as Reinforcement Learning are particularly powerful for discovering optimal topologies for specific mechanical functions [99,100]. However, the most significant advantage cited across nearly all model types is the dramatic computational speedup. As detailed in Table A2, models are often reported to be several orders of magnitude faster than the FEM or RCWA simulations they are trained on, reducing prediction times from hours to milliseconds. This efficiency enables rapid design exploration and optimization. Other benefits highlighted in Table A2 include the ability of deep learning models to handle complex nonlinear relationships and the capacity for generative models to perform automated inverse design.

In summary, the selection of a specific ML architecture must be aligned with the geometric representation of the metamaterial and the design objectives. DNNs are suitable when the design is represented via a small set of geometry parameters [101]. However, they are highly susceptible to overfitting when trained on small datasets and cannot be easily scaled to complex topologies. As was mentioned in the previous subsection, CNNs are the choice for 3D grids, which are common outputs of density-based topology optimization. Nevertheless, they are highly sensitive to voxel resolution. If it is increased, the amount of computing and memory occupied by 3D convolution will also increase. GNNs are powerful models, treating joints as nodes and struts as edges [61]. Meanwhile, they require highly specialized data preprocessing to construct graph topologies. The problem might also be to model continuous boundaries or complex curved shell surfaces. GANs and VAEs are suitable for the exploration of design spaces and discovering novel geometries [65,96]. The limitation is that training might be unstable, which may generate repetitive, structurally identical designs. The models also struggle to enforce strict structural connectivity [65]. This may lead to occasionally producing physically disconnected floating features.

## 4. Applications in Metamaterial Design and Performance Analysis

Having outlined the primary ML models in Section 3, this section examines their practical use and effectiveness in the context of mechanical metamaterial design. It presents how these models have been effectively applied to forward property prediction and inverse design tasks in mechanical metamaterial design, emphasizing their performance as measured by standard evaluation metrics.

### 4.1. Methodologies for Performance Evaluation

To quantitatively assess and compare the performance of ML models, a set of standard evaluation metrics is consistently employed. For regression tasks, which are dominant in property prediction, the most common metrics are the Mean Absolute Error (MAE) and the Mean Squared Error (MSE). Both quantify the average magnitude of error between the model’s predictions and the ground-truth values, with MSE more heavily penalizing larger errors. The Coefficient of Determination (R^2^) is also crucial, as it measures the proportion of the variance in the output that is predictable from the input.

Variations of these metrics, such as the Root Mean Square Error (RMSE) and the Mean Absolute Percentage Error (MAPE), are also frequently used. For instance, in a study optimizing a Long Short-Term Memory (LSTM) network to predict the bandwidth of metamaterial antennas, RMSE and MAPE were used to demonstrate that the optimized model achieved significantly lower prediction errors compared to its standard counterpart [102]. In addition to predictive accuracy, computational efficiency—often measured by the time required for training or prediction—is a critical performance indicator, as a key motivation for using ML is to accelerate the design process compared to traditional, computationally expensive simulations like FEA [65].

### 4.2. Applications in Forward Prediction of Mechanical Properties of Metamaterials

A well-established use of ML in this domain involves increasing the forward prediction of mechanical properties. In this context, ML models are trained to learn the complex relationship between a metamaterial’s geometry and its physical response, serving as a rapid surrogate for computationally expensive FEA simulations [61,65,82,86]. As summarized in Table A1, a variety of models have demonstrated exceptional performance in this area, achieving high accuracy in predicting properties ranging from linear elasticity to complex, nonlinear behaviors.

FNNs and DNNs, for instance, have been widely applied to forward prediction tasks due to their versatility. They have proven effective in predicting the effective mechanical properties, such as Young’s modulus and Poisson’s ratio [103,104], for various lattice structures with high fidelity [65]. A residual fully connected neural network was used to design metamaterial perfect absorbers and predict optical properties, achieving high-accuracy absorption performance [105]. Additionally, the performance of these models in one study, highlighted in Table A1, reported achieving R^2^ scores of 0.987 for modulus and 0.994 for Poisson’s ratio using a neural network, indicating an excellent model fit.

For designs represented by grid-based data, such as 3D voxels, CNNs are particularly useful. By automatically extracting spatial features from the input geometry, CNNs can directly predict complex properties. For example, several 3D CNNs were used to predict the full elasticity tensor of a metamaterial from its voxelized representation [65]. This approach bypasses the need for manual feature engineering and provides a holistic understanding of the material’s anisotropic behavior.

The success of these models is consistently validated by high-performance scores across numerous studies. As detailed in Table A1, R^2^ values for property predictions frequently exceed 0.95, confirming that ML models can explain the vast majority of the variance in the mechanical response [61,82]. This high level of accuracy, combined with prediction times that are orders of magnitude faster than conventional simulations, shows the transformative impact of ML on the analysis and characterization of mechanical metamaterials.

### 4.3. Applications in Inverse Design and Generative Discovery

While forward prediction accelerates the analysis of existing designs, the ultimate goal in metamaterial engineering is often inverse design: the automated generation of novel structures that exhibit specific, predefined properties. ML, particularly using generative models and advanced optimization strategies, has been beneficial in this case. As evidenced by numerous studies in Table A1, ML frameworks are now capable of navigating vast and complex design spaces to discover high-performance metamaterials.

GANs and the newer diffusion models are key contributors to progress in this domain. These models can learn the underlying distribution of a given class of metamaterials and then generate new instances with tailored functionalities [106,107]. For example, a constrained generative inverse design network has been used to create customizable metamaterials with a desired strain-dependent Poisson’s ratio, a highly nonlinear and targeted response [108]. Similarly, diffusion models have been employed in a data-driven framework to inversely design energy-absorbing metamaterials, demonstrating their power in creating structures optimized for a specific function [109].

The inverse design paradigm has been successfully applied to a wide range of target properties and material systems. Deep learning was used to inversely design TPMS-based piezoelectric metamaterials [110] and to generate 3D sequential metamaterials that achieve extreme stiffness [111]. The versatility of these methods allows for the design of not just single-function materials but also complex, multi-functional systems. Furthermore, an ML-accelerated framework was developed for the inverse design of programmable, bi-functional metamaterials that can achieve distinct mechanical responses under different loading conditions [112]. Beyond designing for multiple physical functions, a critical step is incorporating practical engineering constraints. A key case study demonstrates this by integrating multi-objective optimization with a generative model to balance performance against manufacturability and compactness [113]. The framework’s RCGAN-MO architecture first performs an inverse design using a GAN, then refines the output with an optimizer controlled by a tunable weight vector. This allows for the customization of the optimization process, where users can assign priority to conflicting objectives like manufacturability or compactness. As a result, a set of distinct architectures can be generated for a single performance target, each optimized for a different balance of practical considerations.

ML and physics-based validation remain tightly linked in these workflows. The process often involves an ML model proposing a candidate design, which is then rapidly evaluated using FEA. This iterative loop, sometimes enhanced with evolutionary algorithms or reinforcement learning, allows for the continuous refinement and optimization of the generated structures. This approach has been used to design metamaterials with targeted nonlinear deformation responses [86] and to discover optimal topologies for properties like tunable bandgaps [95].

### 4.4. Physics Integration

Traditional data-driven ML models can be viewed as similar to phenomenological models [114,115]. These models are built based on empirical data (measurements, observations, etc.) without the need to explain the phenomenon or with little theory behind. Therefore, they are limited in their ability to represent the physical processes. This limitation is described as mechanism uncertainty, which refers to uncertainty about the phenomena (physical processes) responsible for the patterns learned by an ML model.

Relying only on data-driven predictions can cause different risks. These include, for example, a lack of physical consistency [116]. In complex systems, such as metamaterials, it can potentially lead to physically unrealistic predictions or generate structurally unstable designs during inverse design.

Therefore, to reduce mechanism uncertainty and improve the physical reliability of ML models, Physics-Informed Machine Learning (PIML) can be used [116]. Physical domain theory can be integrated into ML through embedding physical laws (such as governing equations from solid mechanics) in the model’s loss function, embedding hard structural or parameter constraints, or incorporating physical principles directly into the model architecture. It allows the model to reflect physical behavior and better capture complex structural responses.

Nowadays, PIML models are increasingly being applied to modeling materials, processes, and related engineering issues. For example, the mechanics-informed HyperCAN framework, including physical constraints to model the behavior of truss lattices, is presented in [117]. A physics-based ML framework, combining physical stress–strain measurements with microstructural mechanics, was applied to execute the finite-strain inverse design of spinodal metamaterials [118]. By using physical knowledge in the learning process, PIML models are able to enhance the reliability of ML-based designs. This reduces the gaps of purely data-driven approaches and contributes to the generation of more reliable and physically valid metamaterial designs.

## 5. Challenges, Limitations, and Future Directions

The successful applications reviewed in the previous sections highlight the transformative potential of ML in metamaterial design. However, the widespread adoption and continued advancement of these data-driven methods face several significant challenges.

### 5.1. Key Challenges and Limitations

Despite its potential, the ML-driven design approach is confronted by challenges related to data, model robustness, and practical implementation. A primary challenge is data availability and quality. The performance of deep learning models is fundamentally dependent on large, diverse, and high-fidelity datasets [61,90]. Additionally, according to Table A2, most models strongly depend on datasets for initial training. Generating such datasets, whether through high-fidelity FEA simulations or physical experiments, is often computationally expensive and time-consuming. This problem can limit the scope of trainable models and may not fully capture the complex nonlinearity and anisotropy of mechanical metamaterials [65].

A second major challenge is model generalizability and interpretability. Many models are trained on narrow datasets representing a specific class of metamaterials, which can lead to overfitting and limit their ability to generalize to new, unseen architectures [91,119]. The patterns observed in Table A2 further support this trend, showing that most reported models remain specialized for a single purpose, for example, predicting microwave performance [120] or designing materials with a specific tunable Poisson’s ratio [121].

Another issue is that many deep learning models lack transparency, making it difficult to trace the logic behind proposed designs. For engineering applications where safety is critical, the lack of transparency can be a major obstacle, given the need to comprehend the design’s physical foundations. Ensuring that models learn the true physics of the system, rather than merely capturing spurious correlations in the data, is an active area of research, with physics-guided approaches showing promise [80].

Finally, significant challenges remain in computational cost and integration with manufacturing. While ML models are fast at inference, training them—especially complex generative models or reinforcement learning agents—can be computationally intensive, requiring significant hardware resources [86]. Moreover, there is often a gap between the intricate, idealized geometries generated by ML algorithms and what is practically achievable with current additive manufacturing technologies. Future frameworks must increasingly incorporate manufacturing constraints directly into the design process to ensure that the generated metamaterials are not only optimal in simulation but also physically realizable [122,123].

Recent work, however, demonstrates how these limitations can be overcome, with the dynamic optimization of a tissue scaffold for a sheep mandible reconstruction serving as a compelling case study of the complete development process in a practical biomedical application [124]. In this study, an ML framework combining neural networks and a genetic algorithm was used to generatively design a functionally graded lattice scaffold tailored to a specific bone defect. This AI-optimized digital design was physically realized by 3D printing a custom-fit implant, which was then assembled with a cadaveric sheep mandible for mechanical validation.

This shows that a computer-designed model can be successfully manufactured and tested, demonstrating that ML-driven design can generate patient-specific products that are not only computationally optimized but also feasible to manufacture.

### 5.2. Future Research Directions and Opportunities

**Figure 5:**
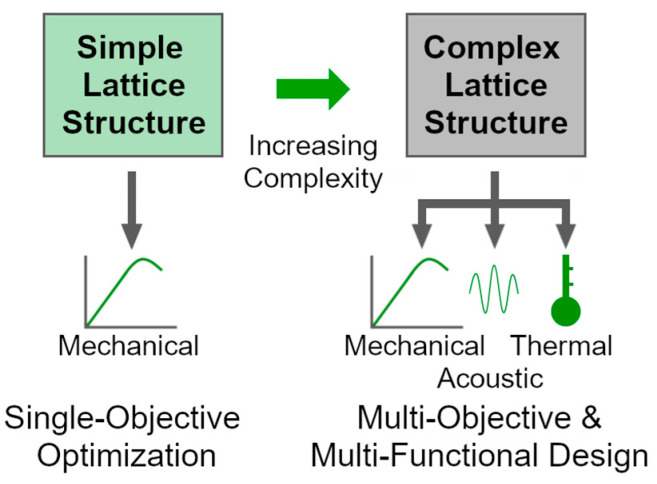
The progression from single-objective optimization for a single property (e.g., stiffness) to multi-objective frameworks that design complex, multifunctional materials with different properties simultaneously.

The outlined challenges serve as a foundation for exploring new opportunities. The growing integration of ML with materials science holds the potential to drive significant advancements and change the limits of metamaterial design. One of the most significant trends is the shift towards designing multifunctional metamaterials. While much of the current research focuses on optimizing a single mechanical property, future work will increasingly target materials that exhibit tailored responses across multiple physical domains simultaneously, such as combining extreme stiffness with specific acoustic or thermal properties [111,125]. This requires the development of multi-objective optimization frameworks capable of navigating the complex trade-offs between competing performance goals (see schematic Figure 5).

Another important point is the multi-scale modeling of multifunctional materials and fusion into a single usable model. This incorporates micro-, meso-, and macro-scales. Instead of studying metamaterial unit cells separately, future research can focus on applying ML surrogate models to structural optimization. One way of applying such an approach is to use ML to optimize topology while maintaining connectivity between neighboring cells [126]. In addition, the developed models can connect microstructure and manufacturing defects with unit cells and overall engineering structures. Meanwhile, the resulting solution may include several approaches, such as ML, PIML, FEA, and topology optimization.

**Figure 6:**
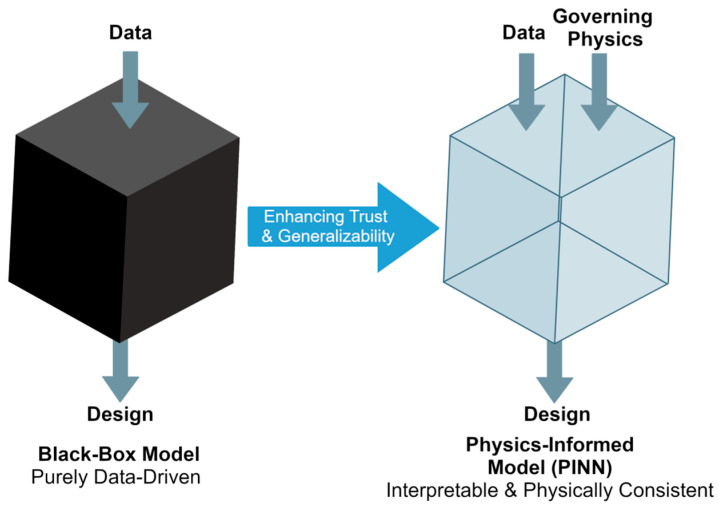
The conceptual evolution of data-driven “black-box” models to transparent “glass-box” Physics-Informed Neural Networks (PINNs).

Another key direction involves developing models that are both interpretable and aligned with physical principles, with a growing emphasis on transparency and trust in model behavior. This includes the use of interpretable ML techniques that can explain the reasoning behind a model’s design choices, bridging the gap between data-driven discovery and physical intuition [127]. A powerful approach in this area is the use of Physics-Informed Neural Networks (PINNs), which embed the governing physical equations (e.g., the differential equations of mechanics) directly into the learning process. This ensures that the model’s predictions are physically consistent, reduces the reliance on large datasets, and improves generalizability [128]. The conceptual evolution of data-driven “black-box” models to transparent “glass-box” Physics-Informed Neural Networks (PINNs) is illustrated in Figure 6 [129,130]. These models can enhance trust and generalizability by making their internal logic interpretable and physically consistent. Future work should focus more on complex 3D structures, incorporating more complex mechanical behavior, such as nonlinearity, plasticity, failure, and so on. For example, in one of the recent studies, the PINN framework was developed for conical Kresling origami design, allowing for a data-free approach for programming complex mechanical behavior [131].

**Figure 7:**
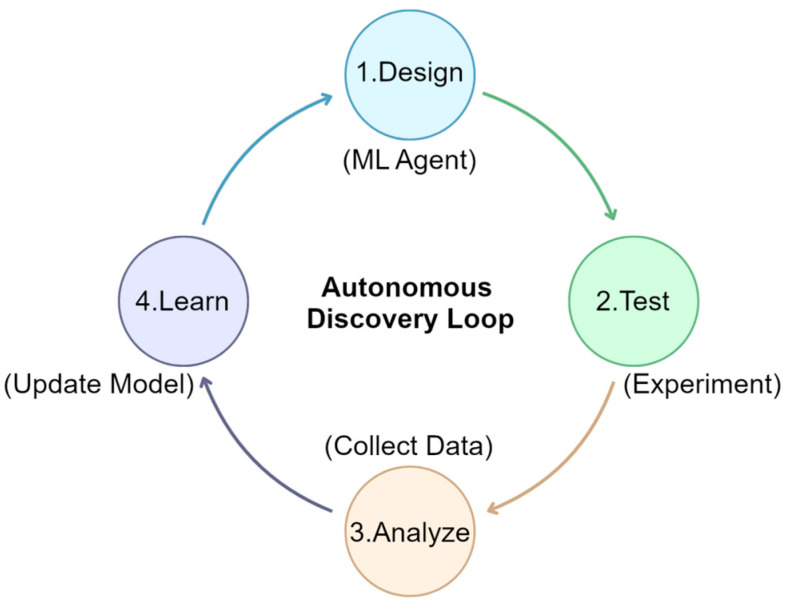
The conceptual workflow of an autonomous “self-driving” laboratory, where an ML agent designs, tests, analyzes, and learns from results in a closed loop to accelerate the pace of discovery.

A long-term goal in the field is the creation of autonomous discovery systems. These would be fully integrated, closed-loop platforms where ML algorithms not only propose novel metamaterial designs but also control the robotic experiments or simulations needed to test them. By continuously learning from the results, these autonomous systems could independently explore the design space, identify promising new materials, and accelerate the pace of discovery far beyond what is currently possible. The integration of reinforcement learning agents into these workflows is a critical step toward realizing this goal of a “self-driving laboratory” for metamaterials [86], as depicted in Figure 7. The future can potentially focus on active learning, with a priority on the most informative designs and maximizing learning efficiency. The built simulation and testing systems can provide the result verification. The framework needs to adapt when differences between experimental and forecast results occur, and correct the design process correspondingly.

Finally, there are opportunities in interdisciplinary approaches. As discussed above, PIML already represents a fusion of machine learning and physical principles. Future developments could further incorporate nonlinear mechanics, plasticity, fatigue behavior, etc. The integration of microstructural information fosters a combination of materials science and data science. Also, ML integrated into metamaterial manufacturing processes enables better characterization of process-induced defects. Applying such interdisciplinary approaches together would enable taking into account material properties, structural mechanical behavior, and manufacturing imperfections, fostering the development of novel metamaterials with improved performance.

## 6. Conclusions

The integration of ML with the design of mechanical metamaterials signifies a transformative shift in material engineering, enabling the creation of innovative structures with unprecedented properties. This review has provided a comprehensive overview of this rapidly advancing field, from the foundational principles of data preparation and the design workflow to the specific ML architectures being employed. The exploration of diverse architectures, from strut-based lattices to triply periodic minimal surfaces, has illuminated the vast potential for achieving unique behaviors such as negative Poisson’s ratios and tunable stiffness.

Through the application of advanced ML techniques, it became possible to accelerate the design cycle dramatically. As demonstrated by a review of recent applications, ML models can accurately predict mechanical properties in a fraction of the time required by traditional simulations and can autonomously generate novel designs through inverse design frameworks. The interaction between data-driven exploration and physics-based validation forms an approach that is driving progress in material discovery. Furthermore, generative models are paving the way for the exploration of complex design spaces that were previously inaccessible.

Despite ongoing difficulties related to data, interpretability, and manufacturing, the future of the field holds great potential. Emerging trends towards multifunctional materials and autonomous discovery systems promise to address these problems and unlock even greater capabilities. As material design continues to evolve, the integration between ML and mechanical metamaterials is deepening our understanding of material behavior and offering significant potential for practical applications in different industries. The ongoing research in this field is expected to produce increasingly advanced materials designed for targeted functions, potentially driving breakthroughs that reshape traditional approaches in materials science.

## Figures and Tables

**Table 1:** Comparison of ML paradigms for metamaterial design, rated by their effectiveness in property prediction and practical feasibility. Ratings are based on typical performance reported in the literature, availability of labeled data, algorithm maturity, and ease of integration into metamaterial design workflows.

Paradigm	Description	Property Prediction Performance	Implementation Feasibility
Supervised Learning	Learns from labeled data	■■■■■	■■■■□
Unsupervised Learning	Finds patterns in unlabeled data	■■□□□	■■□□□
Reinforcement Learning	Explores via reward feedback	■■■■□	■■□□□
Semi-supervised learning	Learns from labeled and unlabeled data	■■■□□	■■■□□

## Data Availability

No new data were created or analyzed in this study. Data sharing is not applicable to this article.

## Appendix A

**Table A1:** Comparative summary of recent ML applications in metamaterial design.

ML Model	Dataset	ML Task Type	Evaluation Metric	Score	Ref.
DNN	Size: 7358 samp. (RCWA-generated spectra); 70% train., 20% valid., 10% test.	Inverse Design + Optimization	MAPE, MAE	96% Accuracy, 0.75 s runtime for 737 test samples	[91]
	Size: 23000 samp. Source: FDTD simulations.	Forward Prediction + Inverse Design	MSE, Prediction time (milliseconds)	MSE: 0.0002, IDNN MSE: 0.00011 (with transfer learning), prediction time: 3.9 ms (forward prediction), 5.6 ms (inverse design)	[132]
	Dataset not explicitly mentioned.	Forward Prediction + Inverse Design + Optimization	MSE, Prediction time (milliseconds)	Test MSE: 0.00087 (before modification), 0.00080 (after AN correction), Prediction time: 9 ms (inverse retrieval), <1 s (training prediction)	[133]
	Source: Simulated data representing optical responses of interlayer-coupled chiral metamaterials. Size: Not explicitly mentioned.	Inverse Design	Comparison between simulated and predicted spectra.	High accuracy in spectral prediction, with simulated results matching original spectra.	[134]
	Source: Simulated data from isogeometric analysis (IGA) for tetra-chiral auxetic struct. Size: 10,000 samp.; 7000 train., 3000 valid., 500 test.	Inverse Design	MSE	Train. MSE: 0.000327 Valid. MSE: 0.000589 Test. Accuracy: Average relative error of 3.8% for Poisson’s ratio, 0.8% for Young’s modulus	[104]
FNN	Size: 20,000 samples; 70% train., 20% valid., 10% test. Source: FEA simulations	Forward Prediction + Inverse Design + Optimization	MSE (model training). R^2^ Score (prediction accuracy).	MSE: 0.0012 (modulus), 0.0008 (Poisson’s ratio), 0.0009 (volume). R^2^ Scores: 0.987 (modulus), 0.994 (Poisson’s ratio), 0.991 (volume).	[135]
	Size: 3780 total spectra (420 spectra per sample); approx. 76% train., 24% test. Source: RCWA simulations.	Forward Prediction + Optimization	MSE, Pearson Correlation Coefficient (R-value).	MSE Loss: 0.025 (training), 0.026 (testing). R-value: >0.90 for 83% of test samples. Computation Speedup: 4 orders of magnitude faster than RCWA.	[136]
	Source: Simulated absorption spectra of chiral plasmonic metamaterials using FDTD. Size: 3900 samples.	Forward Prediction and Inverse Design	Forward Prediction: R^2^ score, RMSE, MAE. Inverse Design: Comparison between simulated and predicted responses.	Forward Prediction: R^2^ = 0.943–0.995, RMSE = 0.006–0.013, MAE = 0.004–0.009	[137]
MLP	Size: 20 initial samp., expanded to 400,000 samp. via Bayesian Optimization. Source: FEA simulations.	Optimization	Stiffness Ratio Metrics (non-reciprocity and asymmetry). Bayesian Optimiz. Performance Measure.	Non-Reciprocity Scores: f1 = 12.75, f2 = 13.68, f3 = 492.50, f4 = 23.95, f5 = 516.00, f6 = 26.80, f7 = 18.67, f8 = 43.56. Elastic Asymmetry Scores: g1 = 2.02, g2 = 492.50, g3 = 27.48, g4 = 1.01, g5 = 1.20, g6 = 25.00, g7 = 1763.03, g8 = 2.24.	[138]
	Source: Simulated data from CST Microwave Studio (electromagnetic responses of chiral metamaterials). Size: 13,800 samp.; 75% train. 25% test.	Forward Prediction and Inverse Design	MSE	Forward Prediction Accuracy: Test MSE = 1.47 × 10^−4^. Inverse Design Accuracy: Test MSE reduced from 1.52 × 10^−5^ to 9 × 10^−6^ with enhanced model tuning.	[139]
Multiple Linear Regress (MLR), Gaussian Process Regress (GPR), and Polynomial Chaos Expansion (PCE)	Size: 200 samp. (FEM-generated Poisson’s ratio). Experimental validation with 196 converged samp.	Optimization	Leave-One-Out Cross-Validation (LOOCV) error, Fivefold Cross-Validation (5-fold CV) error	LOOCV error (Poisson’s ratio prediction)-GPR: 0.0382, PCE: 0.0356, MLR: 0.1506. LOOCV error (Porosity prediction)-GPR: 0.0032, PCE: 0.0023, MLR: 0.0269.	[140]
Artificial Neural Network (ANN), Random Forest Regress (RFR), Support Vector Regress (SVR) with Model-Agnostic Data Enhancement (MADE)	Size: 1899 target samples + 2785 target samples. Source: RCWA + additional MADE-enhanced dataset	Forward Prediction	MAE, MAPE, Computational Time (CPU Time per 100 samples)	MAE (First Category Metamat., 1200 Samp.): ANN: 1.399 × 10^−3^; RFR: 1.694 × 10^−3^; SVR: 14.392 × 10^−3^. MAE (Second Category Metamat, 2000 Samp.): ANN: 1.649 × 10^−3^; RFR: 7.919 × 10^−3^; SVR: 24.042 × 10^−3^. MAPE (First Category Metamat): ANN: 0.676%; RFR: 0.73%; SVR: 4.806%. CPU Time per 100 Samp. (First Category): ANN: 5.52 × 10^−3^ ms; RFR: 2.09 ms; SVR: 0.46 ms	[141]
FNN, DNN, GA for hyperparameter optimization	Training dataset size unspecified, model trained on RCWA-generated optical chirality spectra.	Forward Prediction + Optimization	MAE, MAPE, R-value, Computational Time (CPU Time per 100 samples)	MAE: 0.02 (train), 0.03 (test). MAPE: 0.13% (min), 22% (max), 1.07% (mean). R-value: 97% of test cases have R > 99%. Computational Time: 1 ms per 100 samples (FC-NN), 5.5 h per 100 samp. (RCWA)	[142]
Conditional Generative Adversarial Network (c-GAN), Forward Neural Network (F-NN)	Size: 100,000 samp.; 90% train, 10% test. Source: FEA simulations.	Forward Prediction + Inverse Design + Optimization	MAE, R^2^ Score	MAE: Bandgap start freq.: 4.708 Hz, Bandgap end freq.: 12.187 Hz, Max comp stress: 20.248 MPa, Max shear stress: 10.092 MPa. R^2^ Scores: 0.996 (bandgap start), 0.996 (bandgap end), 0.915 (max compress stress), 0.913 (max shear stress)	[143]
MADE	Size: 7000 original sampl. and 40,000 synthetic samp. via MADE; 70% train., 20% valid., 10% test. Source: RCWA simulations.	Forward Prediction + Inverse Design	MAPE for inverse design accuracy. Speedup factor over RCWA simulations.	MAPE: ≤5% across different metamaterial categories. Computation Speedup: 4 orders of magnitude faster than RCWA (inverse design in <1 s).	[144]
Multitask Deep Learning (MDL) Model	Size: 640 samp.; 80% train., 20% test. Source: FEA simulations.	Forward Prediction + Inverse Design + Optimization	MSE (prediction accuracy). Convergence Speed of MDL Training.	MSE Loss: 0.000441. Computation Speedup: 7 orders of magnitude faster than direct simulations.	[145]
CNN	Size: Dataset1: 1210 samp., Dataset2: 2057 samp.; 80% train., 20% test. (with variations in train set sizes: 80%, 50%, 30%, 10%). Source: CST Microwave Studio simulations (FEA-based electromagnetic solver).	Classification (Resonance Frequency Prediction)	Accuracy (for classification performance). Precision, Recall, and F-Measure (for detailed model performance evaluation).	CNN Accuracy: 58.29% (Dataset1, train set 80%), 68.77% (Dataset2, train set 80%). Other Models (Dataset2, train set 80%): RF: 55.84%, SVM: 54.91%, NB: 52.20%, DT: 46.42%. Precision, Recall, and F-Measure: CNN (F-Measure: 76.51, Precision: 73.04, Sensitivity: 80.59).	[146]
Feedforward Neural Network (FFNN)	Source: Simulated data using FEA on chiral metamaterial structures. Size: 190,000 samples; 80% train, 15% valid, 5% for test.	Forward Prediction and Inverse Design	MSE	Forward Prediction MSE: Not explicitly stated, but minimized during training. Inverse Design Accuracy: Verified through FEA and experimental validation.	[147]

**Table A2:** Qualitative comparison of key machine learning architectures for metamaterial design.

ML Model	Advantages	Disadvantages	Ref.
DNN	Reduces Data Requirements for Training. Faster than Traditional Optimization Methods, High Accuracy and Efficiency. Interpretable Predictions via Regressor Chains. Flexible for Different Chiral Metamaterial Structures	Relies on Initial Dataset from RCWA Simulations. Potential Data Distribution Inconsistency. Parameters Can Cause Errors. Requires Hyperparameter Tuning.	[91]
	Fast and efficient forward and inverse design. Improves accuracy using 1D-CNN feature extraction. Reduces data requirements with transfer learning. Applicable to a variety of nanophotonic designs.	Dependence on large datasets for initial training. Lower accuracy for sharp spectral features (Rayleigh anomaly issue). Model complexity increases with deeper architectures.	[132]
	Fast and efficient inverse design, Reduces dependence on numerical simulations. Enables on-demand design of metamaterials. Handles complex nonlinear relationships between structure and response	Dependence on large datasets for initial training. Limited accuracy for sharp resonances. Computational cost for training.	[133]
	Enables efficient inverse design of chiral metamaterials. High accuracy in predicting structural parameters from spectral data. Reduces reliance on manual optimization and trial-and-error design.	Training data requirements: Requires large, high-quality datasets for accurate results. Generalization limitations: May not work well outside trained parameter space. Computational cost: Training deep networks requires significant resources.	[134]
	Significantly faster than conventional IGA-based homogenization methods. Enables real-time inverse design of auxetic structures. Provides explicit analytical gradients, improving optimization efficiency.	Training requires a large dataset, increasing pre-processing time. Inverse design accuracy depends on dataset diversity and model generalization. May struggle with highly nonlinear relationships in mechanical properties.	[104]
FNN	High accuracy in material property prediction. Scalable to different materials and structural configurations. Experimental validation confirms model reliability.	High computational cost of FEM-based dataset generation. Potential overfitting for materials not included in the training set. Limited generalization for extreme design parameters.	[135]
	Significant computation speed improvement. Maintains high accuracy in CD response predictions. Uses Particle Swarm Optimization (PSO) to fine-tune hyperparameters.	Inverse design is not implemented, only forward prediction. Accuracy depends heavily on dataset quality and RCWA simulations. Potential limitations in predicting CD responses for unseen metamaterial configurations.	[136]
	High accuracy in predicting CD response from structural parameters. Reduces the dimensional mismatch between input and output, improving training stability. Permutation importance analysis provides insights into key design parameters.	Inverse design faces challenges with small structural deviations affecting spectral response. Performance depends on dataset quality and generalization outside training conditions. Requires careful tuning of input feature selection for best results.	[137]
MLP	Efficient discovery of optimized chiral structures. Flexible framework for multi-objective optimization. Real-world applicability validated through simulations.	High computational cost of FEA-based data generation. Optimization requires significant tuning for best performance. Limited interpretability of neural network surrogate models.	[138]
	High prediction accuracy for THz responses of chiral metamaterials. Bidirectional model enables inverse design, allowing retrieval of metamaterial structures from target responses. Much faster than traditional trial-and-error approaches for metamaterial design.	Inverse design faces challenges due to one-to-many mapping (multiple structures can yield similar responses). High computational cost for model training and data generation. Potential overfitting issues, requiring careful dataset selection and regularization.	[139]
MLR, GPR, and PCE	Provides accurate design exploration with minimal data. More interpretable results via Explainable ML techniques (SHAP, GSA). Combines ML with FEA simulations for better real-world validation. GPR provides a flexible non-parametric approach, while PCE captures uncertainty in parametric settings.	Limited by the accuracy of FEA-generated training data. Potential overfitting risk due to small dataset (200 samples). Computational cost of hyperparameter tuning in GPR. MLR is too simplistic for capturing complex nonlinear relationships	[140]
ANN, RFR, SVR with MADE	Significantly reduces training data requirements via MADE. Improves ML performance through transfer learning (domain adaptation). Maintains high accuracy with less computational cost compared to full numerical simulations. Flexible and applicable to different ML models (ANN, RFR, SVR).	Higher computational time compared to standalone ML models. Performance depends on the choice of source domain dataset (domain adaptation efficiency). SVR model performs significantly worse than ANN and RFR in MAE and MAPE metrics.	[141]
FNN, DNN, GA	Ultrafast computational speed. Highly accurate predictions (MAPE < 1.07% on average). Optimized hyperparameters using GA. Can study nonlinear dependencies between geometric parameters and CD responses.	No explicit inverse design capability. Performance depends on training dataset quality (RCWA-generated data). Hyperparameter tuning using GA adds computational overhead. Requires large amounts of labeled data for training.	[142]
c-GAN, F-NN	High accuracy in inverse design. Experimental validation. Balances multiple mechanical constraints. Scalability to other metamaterial applications.	High computational cost. Complexity in stress predictions. Potential overfitting risk.	[143]
MADE	Computation speed improvement. Requires fewer training samples due to data augmentation. Multi-task learning improves inverse design accuracy.	Relies heavily on synthetic “pseudo-data” (may introduce noise). Potential generalization issues when extending to completely new chiral designs. Inverse design accuracy is lower for complex structures.	[144]
MDL	Multitask learning improves generalization and efficiency. Avoids high computational cost of full-wave simulations. Fast convergence and highly accurate predictions.	Inverse design accuracy may drop for highly complex structures. Dependent on high-quality training data. Training requires computational resources.	[145]
CNN	CNN outperforms conventional ML models for resonance frequency classification. CNN achieves at least 4% higher accuracy than the best-performing traditional ML model (RF). Deep learning enables better feature extraction compared to conventional approaches.	Lower classification accuracy than some state-of-the-art DL models. Deep learning requires larger datasets to outperform ML models significantly. Generalization issues with limited dataset sizes.	[146]
FFNN	Efficient mapping between structure and mechanical properties. Significantly reduces computational cost compared to traditional FEA. Enables inverse design for precise material properties.	Inverse design is affected by one-to-many mapping issues. High training cost and dataset generation overhead. Generalization is limited outside the trained dataset range.	[147]
